# Association of Timing and Duration of Prenatal Analgesic Opioid Exposure With Attention-Deficit/Hyperactivity Disorder in Children

**DOI:** 10.1001/jamanetworkopen.2021.24324

**Published:** 2021-09-15

**Authors:** Johanne Naper Trønnes, Angela Lupattelli, Marte Handal, Svetlana Skurtveit, Eivind Ystrom, Hedvig Nordeng

**Affiliations:** 1 PharmacoEpidemiology and Drug Safety Research Group, Department of Pharmacy, PharmaTox Strategic Research Initiative, Faculty of Mathematics and Natural Sciences, University of Oslo, Oslo, Norway; 2 Department of Mental Disorders, Norwegian Institute of Public Health, Oslo, Norway; 3 PROMENTA Research Center, Department of Psychology, University of Oslo, Oslo, Norway; 4 Department of Child Health and Development, Norwegian Institute of Public Health, Oslo, Norway

## Abstract

**Importance:**

Prior studies have reported that the use of illicit opioids during pregnancy is associated with increased risk of attention-deficit/hyperactivity disorder (ADHD) in offspring; however, evidence regarding the association of analgesic opioids is limited.

**Objective:**

To examine the association of timing and duration of prenatal analgesic opioid exposure with ADHD in children.

**Design, Setting, and Participants:**

This cohort study uses data from the Norwegian Mother, Father and Child Cohort study (1999-2008), a nationwide birth cohort study linked to national health registries, with a mean (SD) follow-up of 10.8 (2.2) years. A total of 73 784 live-born singleton children born to 62 013 mothers who reported a pain-related condition before and/or during pregnancy were included, with 2 comparator groups: (1) mothers who did not use any opioids and (2) mothers who used opioids before pregnancy only. Data were analyzed from June to December 2020.

**Exposures:**

Maternal self-report of analgesic opioid use during pregnancy, by timing (early and middle and/or late) and duration (≥5 weeks vs ≤4 weeks).

**Main Outcomes and Measures:**

Diagnosis of ADHD or filled prescription for ADHD medication in children and symptoms of ADHD at child age 5 years, measured by Conners’ Parent Rating Scale–Revised. Inverse probability of treatment weights were used to control for measured confounding. Cox regression was used to estimate hazard ratios (HRs) and 95% CIs.

**Results:**

The analyses of ADHD diagnosis and ADHD symptoms included 73 480 children (35 996 [49.0%] girls; mean [SD] maternal age, 30.0 [4.6] years) and 31 270 children (15 377 [49.2%] girls; mean [SD] maternal age, 30.5 [4.4] years), respectively. Overall, 1726 children in the ADHD diagnosis sample (2.3%) and 667 children in the ADHD symptom sample (2.1%) were exposed to an analgesic opioid at least once during gestation. No associations between timing of prenatal analgesic opioid exposure and ADHD diagnosis or symptoms was found. Exposure for 5 or more weeks was associated with an increased risk of ADHD diagnosis (HR, 1.60, 95% CI, 1.04-2.47) compared with exposure for 4 weeks or less; however, there was no such association for the risk of ADHD symptoms.

**Conclusions and Relevance:**

In this cohort study, a slightly elevated risk of ADHD diagnosis after prenatal analgesic opioid exposure for 5 or more weeks was found compared with exposure for 4 weeks or less. This result may be driven by longer duration of use; however, the role of residual or unmeasured confounding cannot be excluded. This finding needs to be replicated in other studies.

## Key Points

**Question:**

Is prenatal analgesic opioid exposure associated with attention-deficit/hyperactivity disorder (ADHD) in children?

**Findings:**

In this cohort study of 73 480 children, with a mean follow-up of 11 years, no association between timing of analgesic opioid exposure during pregnancy and ADHD was found. The risk of ADHD diagnosis was elevated after exposure to opioids for 5 or more weeks compared with exposure for 4 weeks or less.

**Meaning:**

The increased risk of ADHD observed in this study may be driven by longer duration of exposure; however, the role of residual or unmeasured confounding cannot be excluded, and this finding requires further study.

## Teaser

This cohort study examines the association of timing and duration of prenatal analgesic opioid exposure with attention-deficit/hyperactivity disorder (ADHD) in children.

## Introduction

Many women experience pain during pregnancy, and although not recommended as the first choice for pain management in pregnancy, opioids are at times prescribed due to their analgesic effect.^1,2^ In recent years, consumption of prescribed opioid analgesics has increased,^3^ a trend also affecting women of childbearing age.^4,5,6,7^ Prevalence estimates among pregnant women range from 1% in multinational surveys^8^ based on maternal self-report to 3% in Norway^9,10^ and 14% to 28% in the United States based on dispensed prescriptions.^5,6,7^

Results from animal studies suggest that prenatal opioid exposure may alter fetal brain structure and functioning, thus potentially interfering with normal brain development.^11,12,13^ However, evidence regarding the long-term consequences of prenatal opioid exposure on child neurodevelopment, including behavioral outcomes, is still limited.^14,15,16^

One of the most common behavioral disorders in childhood is attention-deficit/hyperactivity disorder (ADHD), which affects approximately 2% to 7% of children worldwide.^17,18^ The median age of first diagnosis is estimated to be around 7 to 9 years.^19^ The cause of ADHD is multifactorial, and ADHD has been associated with a broad range of negative outcomes later in life.^18^

Associations of prenatal opioid exposure with ADHD have been observed; however, prior studies have mainly been done in selected populations, such as women receiving opioid maintenance treatment or among women with opioid dependence.^20,21^ Azuine et al^22^ reported an odds ratio of 2.55 (95% CI, 1.42-4.57) for ADHD after opioid exposure when children with exposure were compared with those without. Currently, there is a knowledge gap regarding the association of prenatal analgesic opioid exposure with ADHD. This study sought to fill this gap by focusing specifically on women using analgesic opioids for pain management. The aim of this study was to examine the association between timing and duration of prenatal analgesic opioid exposure and (1) ADHD diagnosis and/or filled prescription for ADHD medications and (2) ADHD symptoms at child age 5 years.

## Methods

### Study Population and Data Collection

This study used data from the Norwegian Mother, Father and Child Cohort study (MoBa) (data version 9), the Medical Birth Registry of Norway (MBRN), the Norwegian Prescription Database (NorPD), and the Norwegian Patient Registry (NPR), linked via the woman’s personal identification number and pregnancy sequence (eFigure 1 in the Supplement). The establishment and data collection in MoBa was previously based on a license from the Norwegian Data protection agency and approval from The Regional Committee for Medical Research Ethics (reference No. 2015/442), and it is now based on regulations related to the Norwegian Health Registry Act. The current study was approved by The Regional Committee for Medical Research Ethics Written informed consent was obtained from all participants. The Strengthening the Reporting of Observational Studies in Epidemiology (STROBE) reporting guideline was followed.

MoBa is a prospective population-based pregnancy cohort conducted by the Norwegian Institute of Public Health.^23^ Pregnant women from all over Norway were recruited between 1999 and 2008 through a postal invitation in connection with their routine ultrasonography examination in gestational week (GW) 17 or 18. The initial participation rate was 41%, and the cohort now includes 114 500 children, 95 200 mothers, and 75 200 fathers. Mothers were followed up by paper-based questionnaires during pregnancy (in GW 17 [Q1] and 30 [Q3]) and after the child was born (at 6 months [Q4], 18 months, 3 years, 5 years [Q–5 years], 7 years, 8 years, 13 years, 14 years, and 16 to 17 years).

MBRN includes information on pregnancy, delivery, and neonatal health for all births from GW 12 in Norway.^24^ The NorPD contains information about all prescribed medications (irrespective of reimbursement) to individuals in ambulatory care since 2004.^25^ The NPR contains records on admission to hospitals and specialist health care on an individual level since 2008.^26^ The data include date of admission and discharge as well as primary and secondary diagnosis and cover all government-owned hospitals and outpatient clinics and all private health clinics that receive governmental reimbursement. Diagnostic codes in the NPR follow the *International Statistical Classification of Diseases and Related Health Problems, Tenth Revision *(*ICD-10*).

**Figure 1.**
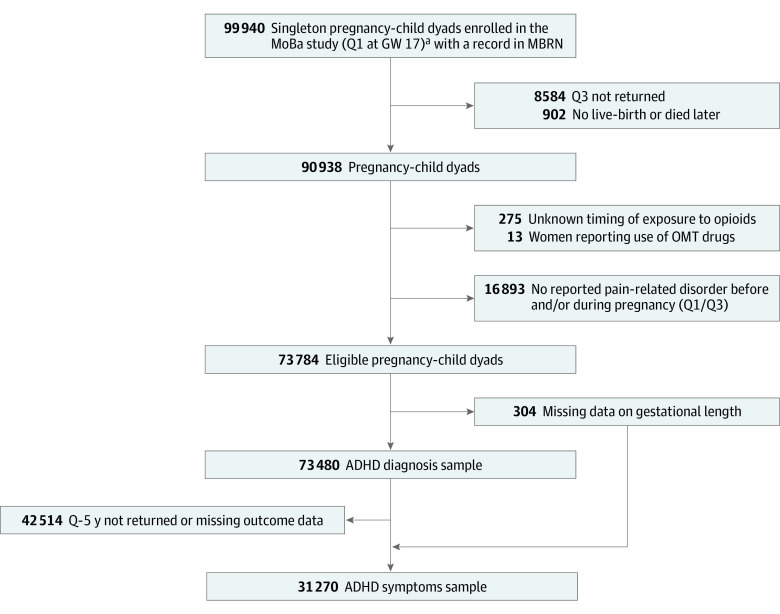
Flowchart to Achieve the Final Study Population ADHD, attention-deficit/hyperactivity disorder; MBRN, Medical Birth Registry of Norway; OMT, drugs used in opioid maintenance therapy (ie, ATC N07BC); Q, questionnaire. ^a^Q1 was the first Norwegian Mother, Father and Child Cohort Study (MoBa) questionnaire completed at gestational week (GW) 17; its completion implies enrollment into the study. Conditions of exclusion can overlap.

We included live-born singletons with a record in MBRN, born to women who completed the 2 prenatal questionnaires (ie, Q1 and Q3) in MoBa. Women with unknown timing of analgesic opioid exposure as well as women who reported a drug used for opioid maintenance treatment were excluded. We restricted the population to women reporting an underlying indication for treatment with analgesic opioids, ie, pain conditions, to emulate the design of a hypothetical clinical trial.^27^ The list of included pain conditions is found in eTable 1 in the Supplement. Figure 1 outlines the inclusion/exclusion criteria to achieve the final study population(s).

### Analgesic Opioid Exposure

Information about medication use was obtained from MoBa Q1, Q3, and Q4. Women reported the name of the medication taken along with timing of use (6 months prepregnancy and during pregnancy by 4-week intervals [eg, GW 0-4, GW 5-8, or GW 9-12]) according to listed indications. All medications were coded according to the Anatomical Therapeutic Chemical (ATC) classification system.^28^ The exposed group included children of women who reported use of analgesic opioids in pregnancy, defined as reporting of ATC code N02A. Individual substances included in the exposure definition appear in eTable 2 in the Supplement.

First, we examined the association of ever use of analgesic opioids in pregnancy and ADHD. Then, we examined the association of timing and duration of analgesic opioid exposure. Timing was categorized as early pregnancy (first trimester) and middle and/or late pregnancy (second and/or third trimester), while duration of exposure was defined according to whether a single interval (use in ≤4 weeks) or multiple 4-week intervals (use in ≥5 weeks) were indicated on the questionnaires.

Among women with pain ailments before and/or during pregnancy, we defined 2 comparison groups; first, a broad group consisting of children of women who did not report use of analgesic opioids (ie, unexposed group) and, second, a narrower comparison group consisting of children of women who used analgesic opioids prior to pregnancy only (prepregnancy users only). The second comparator group was included to minimize residual confounding, given that these children have mothers whose confounder distribution may be more similar to the mothers of children exposed to opioids during pregnancy.

### Outcomes

The primary outcome was child ADHD diagnosis, defined as at least 1 diagnosis of ADHD recorded in the NPR (*ICD-10* code, F90) from 2008 to 2015 and/or at least 1 filled prescription for an ADHD medication (ie, methylphenidate, atomoxetine, racemic amphetamine, dexamphetamine, and lisdexamphetamine) in NorPD between 2004 and 2016. The *ICD-10* code F90 (hyperkinetic disorder) requires the combination of both inattentive and hyperactive symptoms. The drugs listed are licensed in Norway and used for treatment of ADHD. Diagnosis and treatment are started in the specialist health care service, and 80% of children with an ADHD diagnosis receive pharmacotherapy. There is no lower age limit for receiving a F90 diagnosis; however, it is rare in children younger than 5 years.^17,29,30^ Most children in MoBa were born in 2004 or later, and children born from 1999 to 2003 have available outcome data from age 4 years at the latest (eFigure 1 in the Supplement).

To identify children with difficulties but who did not meet the diagnostic criteria for ADHD, we used a secondary outcome of parent-reported symptoms of ADHD in children at age 5 years. This was measured by 12 items from the Conners’ Parent Rating Scale–Revised Short Form (CPRS-R) included in the MoBa questionnaire at Q–5 years. Thus, the sample was further restricted to those with available outcome data at 5 years. Mean scores were calculated and standardized. Higher *z* scores indicated more symptoms of ADHD. More information is provided in the eMethods in the Supplement.

### Potential Confounding Factors

A large number of factors may be associated with opioid use during pregnancy as well as ADHD, and these were examined with the aid of a directed acyclic graph (eFigure 2 in the Supplement).^31,32,33^ We included the following covariates in our analyses: maternal age, marital status, maternal education, maternal income, parity, prepregnancy body mass index (BMI; calculated as weight in kilograms divided by height in meters squared), folic acid supplement, smoking habits, alcohol use, illicit drug use, maternal chronic conditions in early pregnancy, symptoms of anxiety and depression (measured by a short version of the Hopkins Symptoms Checklist^34^ in Q1), number of pain episodes and comedications during pregnancy, and familial risk of ADHD (addressed by information about maternal and paternal filled prescriptions for ADHD medication). Additional factors (eg, child and paternal characteristics, maternal ADHD traits [Adult ADHD Self-report Scale]) were considered under alternative model specifications (eTable 3 in the Supplement). More details on covariates are given in the eMethods in the Supplement.

### Statistical Analysis

To account for measured confounders, we used propensity score (PS)–based methods with inverse probability of treatment weights (IPTW).^35^ The PS was estimated by a logistic regression model. First, we estimated the probability of ever exposure to analgesic opioids during pregnancy, relative to no exposure, given the previously mentioned confounders. In the analysis by timing of exposure, we estimated the probability of analgesic opioid exposure in early and middle and/or late pregnancy, relative to no exposure in the time window or among those who used opioids before pregnancy only, conditional on the previously mentioned confounders. In analysis by duration of exposure, we estimated the probability of exposure for 5 or more weeks relative to exposure for 4 or fewer weeks, conditional on the previously mentioned confounders. Then, we derived stabilized IPTWs for all comparisons. We could not fairly compare those with opioid exposure for 5 or more weeks with those with no exposure or prepregnancy use due to a large imbalance in covariates. The balance of the covariates was assessed by standardized mean differences, with 0.15 as cutoff for evidence of imbalance.^36,37^ When we were not able to achieve a standardized mean difference less than 0.15 between covariates in weighted populations, the covariates were added to the final weighted model. Characteristics of the weights are presented in eTable 4 in the Supplement. The PS and subsequent weights were estimated in each imputed data set to obtain exposure effect estimates in each imputation and then combined to produce an overall estimate.^38,39^

To estimate the hazard ratio (HR) for ADHD, we performed crude and weighted Cox regression analysis with robust standard errors. We used child age in years as the time scale and a quadratic term for year of birth. The follow-up period started at birth and ended on the date of ADHD diagnosis, date of first drug prescription for ADHD, or December 31, 2016, whichever came first. To estimate standardized mean differences in ADHD symptoms, we fit crude and weighted generalized linear models with robust standard errors. Statistical significance was set at *P* < .05, and all tests were 2-tailed. All statistical analysis were performed using Stata MP version 16.1 (StataCorp). Data were analyzed from June to December 2020.

#### Sensitivity Analyses

We performed several subgroup and sensitivity analyses. First, we conducted separate models for all exposure definitions that considered additional parental and child factors under alternate model specifications (eTable 3 in the Supplement). Second, we performed stratified analysis by child sex, with ever or never exposure to analgesic opioids in pregnancy to better understand the association of child sex with ADHD risk. Third, we performed a positive control analysis among women using opioid cough medications (ATC, R05D) during pregnancy. A commonly used opioid in Norway is the combinatory product of codeine and paracetamol, and we performed an analysis among women using opioids not in combination with paracetamol. To evaluate unmeasured confounding, we calculated the E value, ie, the minimum strength of an unmeasured confounder would need to have with both the exposure and the outcome to account for the association.^40^ In a subsample of women with data available in both MoBa and NorPD (2004-2009), we crosschecked maternal self-reported opioid use with NorPD data and looked at the average defined daily dose (DDD) dispensed to describe the amount of opioids. More information and additional sensitivity analyses are presented in the eMethods in the Supplement.

#### Missing Data and Multiple Imputation

Pattern of missingness was explored, and nearly 20% of the pregnancies had missing values in at least 1 of the sufficient confounders. Under the assumption that data were missing at random, we imputed incomplete data via multiple imputation with chained equation (10 replications).^41,42^ Information on missing values on covariates and the imputation procedure is provided in the eMethods in the Supplement.

## Results

We had 73 480 children of 61 753 mothers with data on ADHD diagnosis (ADHD diagnosis sample; 35 996 [49.0%] girls; mean [SD] maternal age, 30.0 [4.6] years) and 31 270 children of 26 017 mothers with data on ADHD symptoms (ADHD symptoms sample; 15 377 [49.2%] girls; mean [SD] maternal age, 30.5 [4.4] years) (Figure 1). Most children in the ADHD symptoms sample were also included in the ADHD diagnosis sample. Of these children, 1726 in ADHD diagnosis sample (2.3%) and 667 in ADHD symptoms sample (2.1%) were exposed to an analgesic opioid at least once during gestation. The dominating substance was codeine in combination with paracetamol, reported in approximately 90% of the exposed pregnancies. The other substances reported were mainly strong opioids (eTable 2 in the Supplement). The main pain conditions reported among analgesic opioid users were headache or migraine (751 of 1726 [43.5%]), back pain (741 [43.0%]), and pelvic girdle pain (401 [23.2%]). Most women had a college or university education, but mothers of children with exposure were more likely to report smoking, alcohol, and use of comedications during pregnancy. Further characteristics are presented in Table 1 and eTable 5 in the Supplement.

**Table 1.** Characteristics of 73 480 Pregnancies in the ADHD Diagnosis Sample According to Exposure Status

Characteristic	Individuals in ADHD diagnosis sample by opioid exposure, No. (%)		
	No exposure (n = 70 916)	Exposure (n = 1726)	Prepregnancy exposure only (n = 838)
Maternal characteristics			
Age at time of delivery, mean (SD), y	30.0 (4.5)	30.4 (4.6)	29.6 (4.8)
Married or cohabiting	68 169 (96.1)	1636 (94.8)	795 (94.9)
Primiparous	31 972 (45.1)	694 (40.2)	463 (55.3)
Education			
University or college education	47 108 (66.4)	1050 (60.8)	495 (59.1)
Missing	311 (0.4)	5 (0.3)	3 (0.4)
Gross yearly income^a^			
Average	48 424 (68.3)	1137 (65.9)	569 (67.9)
Low	12 536 (17.7)	369 (21.4)	176 (21.0)
High	7585 (10.7)	163 (9.4)	75 (9.0)
Missing	2371 (3.3)	57 (3.3)	18 (2.2)
Prepregnancy BMI, mean (SD)	24.1 (4.3)	25.1 (4.9)	24.8 (4.7)
Missing, No. (%)	1761 (2.4)	21 (2.4)	41 (2.5)
Folic acid supplement	54 630 (77.0)	1275 (73.9)	671 (80.1)
Smoking^b^			
No	53 927 (76.0)	1163 (67.4)	555 (66.2)
Yes	5797 (8.2)	232 (13.4)	121 (14.4)
Stopped	10 343 (14.6)	313 (18.1)	157 (18.7)
Missing	849 (1.2)	18 (1.1)	5 (0.7)
Alcohol intake^b^			
No or minimal	61 464 (86.7)	1441 (83.5)	727 (86.8)
Low to moderate	1671 (2.4)	55 (3.2)	21 (2.5)
Frequent	58 (0.1)	3 (0.2)	0
Missing	7723 (10.9)	227 (13.2)	90 (10.7)
Symptoms of anxiety/depression, mean score (SD)^c^	1.3 (0.4)	1.4 (0.5)	1.4 (0.5)
Missing, No. (%)	2473 (3.4)	72 (4.1)	28 (3.3)
Chronic health conditions^d^	8841 (12.5)	330 (19.1)	166 (19.8)
Comedications during pregnancy^e^	37 986 (53.6)	1476 (85.5)	541 (64.6)
Illicit drug use^b^	135 (0.2)	11 (0.6)	9 (1.1)
ADHD prescriptions^f^	768 (1.1)	48 (2.8)	21 (2.5)
Child characteristics			
Boys	36 185 (51.0)	879 (50.9)	420 (50.1)
Girls	34 731 (49.0)	847 (49.1)	418 (49.9)
Preterm (<37 weeks)	3060 (4.3)	109 (6.3)	44 (5.3)
Low birth weight (<2500 g)	1713 (2.4)	57 (3.3)	18 (2.2)
All malformations	3317 (4.7)	81 (4.7)	37 (4.4)
Paternal characteristics			
Age, y			
<25	3492 (4.9)	76 (4.4)	58 (6.9)
25-29	16 571 (23.4)	396 (22.9)	217 (25.9)
30-34	27 459 (38.7)	655 (37.9)	288 (34.4)
≥35	23 216 (32.7)	593 (34.4)	273 (32.6)
Missing	178 (0.3)	6 (0.4)	2 (0.2)
Education			
University or college education	35 697 (50.3)	769 (44.6)	368 (43.9)
Missing	746 (1.1)	14 (0.8)	14 (1.7)
ADHD prescriptions (%)^f^	536 (0.8)	28 (1.6)	7 (0.8)

Abbreviations: ADHD, attention-deficit/hyperactivity disorder; BMI, body mass index (calculated as weight in kilograms divided by height in meters squared).

^a^ Gross yearly income was classified as follows: average, $17 450 to $46 540; low, less than $17 450; high, ≥$46 541.

^b^ Measured in the first Norwegian Mother, Father and Child Cohort study questionnaire.

^c^ Measured by a short version of the Hopkins Symptoms Checklist on first questionnaire.

^d^ Chronic health conditions include the following conditions: asthma, diabetes, hypertension, Crohn disease, arthritis, lupus, epilepsy, multiple sclerosis, and cancer.

^e^ Comedications in pregnancy include paracetamol, triptans, anti-epileptics, antipsychotics, antidepressants, nonsteroidal anti-inflammatory drugs, and benzodiazepines and benzodiazepine-like drugs.

^f^ Indicates filled prescriptions for ADHD medication ever in life since 2004.

### ADHD Diagnosis

**Figure 2.**
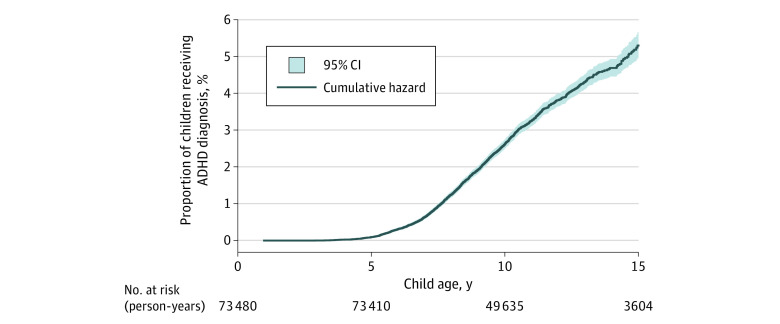
Nelson-Aalen Cumulative Hazard Estimate and the Estimated Proportion of Children Receiving a Diagnosis for Attention-Deficit/Hyperactivity (ADHD) by Child Age

In total, 2211 children (3.0%) had ADHD, and Figure 2 shows its cumulative hazard. Fewer than 5 children were diagnosed before the age of 3 years. The incidence rate was highest at age 7 to 11 years (eTable 6 in the Supplement), and the mean (SD) follow-up time was 10.8 (2.2) years. In crude analysis, ever exposure to analgesic opioids during pregnancy was associated with a higher risk of ADHD (HR, 1.71; 95% CI, 1.38-2.10) compared with no exposure. After weighting, the association was attenuated and no longer statistically significant (weighted HR, 1.32; 95% CI, 0.98-1.76). Exposure in early and middle and/or late pregnancy was associated with a moderate increased risk of ADHD in crude analysis when compared with no exposure in the same time window (Table 2). However, on weighting, the point estimates were attenuated, and the confidence intervals included the null (early exposure: weighted HR, 1.34; 95% CI, 0.90-2.02; middle and/or late exposure: weighted HR, 1.32; 95% CI, 0.92-1.89). No associations were found in analyses comparing analgesic opioid use in early or middle and/or late pregnancy to prepregnancy use only (early exposure: weighted HR, 1.13; 95% CI: 0.71-1.79; middle and/or late exposure: weighted HR, 1.08; 95% CI, 0.70-1.68). Exposure for 5 weeks or more of pregnancy was associated with increased risk of ADHD (weighted HR, 1.60; 95% CI, 1.04-2.47) compared with exposure for 4 or fewer weeks (Table 3).

**Table 2.** Association Between Timing of Prenatal Analgesic Opioid Exposure and ADHD Diagnosis and ADHD Symptoms in Children Aged 5 Years

Exposure window	No.	Events, No.	IR per 1000 person-years	Crude HR (95% CI)	Weighted HR (95% CI)
**ADHD diagnosis sample**					
Exposure vs no exposure					
No opioids in early pregnancy	72 675	2166	2.8	1 [Reference]	1 [Reference]
Opioids in early pregnancy	805	45	5.0	1.76 (1.30 to 2.36)	1.34 (0.90 to 2.02)
No opioids in middle or late pregnancy	72 244	2145	2.8	1 [Reference]	1 [Reference]
Opioids in middle and/or late pregnancy	1236	66	4.9	1.76 (1.38 to 2.25)	1.32 (0.92 to 1.89)
Exposure vs prepregnancy exposure only					
Opioid use in prepregnancy only	838	39	4.2	1 [Reference]	1 [Reference]
Opioids in early pregnancy	805	45	5.0	1.17 (0.76 to 1.80)	1.13 (0.71 to 1.79)
Opioids in middle and/or late pregnancy	1236	66	4.9	1.16 (0.78 to 1.72)	1.08 (0.70 to 1.68)
**ADHD symptoms sample**					
**Exposure window**	**No.**	**Mean**	**SD**	**Crude β (95% CI)**	**Weighted β (95% CI)**
Exposure vs no exposure					
No opioids in early pregnancy	30 973	1.38	0.39	[Reference]	[Reference]
Opioids in early pregnancy	297	1.41	0.42	0.09 (−0.03 to 0.22)	0.08 (−0.08 to 0.24)
No opioids in middle or late pregnancy	30 779	1.38	0.39	[Reference]	[Reference]
Opioids in middle and/or late pregnancy	491	1.40	0.38	0.05 (−0.04 to 0.14)	−0.02 (−0.13 to 0.08)
Exposure vs prepregnancy exposure only					
Opioids prepregnancy only	334	1.43	0.40	[Reference]	[Reference]
Opioids in early pregnancy	297	1.41	0.42	−0.04 (−0.20 to 0.13)	0.05 (−0.14 to 0.24)
Opioids in middle and/or late pregnancy	491	1.40	0.38	−0.08 (−0.22 to 0.07)	−0.02 (−0.19 to 0.16)

Abbreviations: ADHD, attention-deficit/hyperactivity disorder; HR, hazard ratio; IR, incidence rate.

**Table 3.** Association Between Duration of Prenatal Analgesic Opioid Exposure and ADHD Diagnosis and ADHD Symptoms in Children Aged 5 Years

Length of exposure	No.	Events, No.	IR per 1000 person-years	Crude HR (95% CI)	Weighted HR (95% CI)
**ADHD diagnosis sample**					
Exposed in ≤4 weeks	1084	48	4.0	1 [Reference]	1 [Reference]
Exposed ≥5 weeks	642	43	6.2	1.60 (1.06 to 2.41)	1.60 (1.04 to 2.47)
**ADHD symptoms sample**					
**Length of exposure**	**No.**	**Mean**	**SD**	**Crude β (95% CI)**	**Weighted β (95% CI)**
Exposed in ≤4 weeks	423	1.41	0.40	[Reference]	[Reference]
Exposed ≥5 weeks	244	1.40	0.40	−0.01 (−0.18 to 0.15)	−0.05 (−0.25 to 0.15)

Abbreviations: ADHD, attention-deficit/hyperactivity disorder; HR, hazard ratio; IR, incidence rate.

### ADHD Symptoms

We found no associations between ever exposure to analgesic opioids during pregnancy and symptoms of ADHD at child age 5 years (weighted β = 0.03; 95% CI, −0.07 to 0.12) compared with no exposure. No associations were found in analyses of timing or duration (≥5 weeks vs ≤4 weeks: weighted β = −0.05; 95% CI: −0.25 to 0.15) (Table 2 and Table 3).

### Sensitivity Analyses

The point estimates under alternative model specifications were generally consistent with main findings (eFigures 3, 4, and 5 in the Supplement). In analyses stratified by sex, the weighted HRs among boys and girls were similar (boys: HR, 1.28; 95% CI, 0.93-1.77; girls: HR, 1.36; 95% CI, 0.74-2.51). Furthermore, we found no association between sex and ADHD symptoms in children aged 5 years (eMethods in the Supplement).

We found no associations between children exposed to opioid-containing cough medications during pregnancy and ADHD diagnosis (weighted HR, 0.70; 95% CI, 0.47 to 1.05) or symptoms (weighted β = 0.01; 95% CI, −0.10 to 0.12) compared with no exposure. We found no associations between children exposed to analgesic opioids not in combination with paracetamol and ADHD diagnosis (weighted HR, 0.59; 95% CI, 0.23 to 1.53) or ADHD symptoms (weighted β = 0.12; 95% CI, −0.21 to 0.45) compared with no exposure during pregnancy (eMethods in the Supplement). In a subsample of participants with data available in both MoBa and NorPD (50 925 mother-child pairs), the mean (SD) DDDs dispensed among women using opioids for 4 or fewer weeks and 5 or more weeks were 8.6 (8.5) DDD and 37.2 (79.0) DDD, respectively. Results of additional sensitivity analyses are presented in the eMethods in the Supplement (eTables 7, 8, and 9, and eFigure 6, and 7 in the Supplement).

## Discussion

We found no associations between timing of analgesic opioid exposure during pregnancy and ADHD, both as diagnosis and symptoms. The risk of ADHD was slightly increased after exposure for 5 or more weeks compared with exposure for 4 or fewer weeks. However, there was no evidence for such an association in relation to ADHD symptoms in children at age 5 years. Our results may indicate that the increased risk of ADHD could be driven by longer duration of use; however, the role of residual confounding cannot be ruled out.

All women included in the study reported having an underlying indication for treatment with analgesic opioids, ie, pain conditions; however, there is a heterogeneity of pain-related disorders. Therefore, we included a second comparator group consisting of women who used analgesic opioids prior to pregnancy only. The group with prepregnancy opioid use only may be a fairer comparison group, with a more similar confounder structure as women using opioid analgesics in pregnancy because both groups have a history of analgesic opioid exposure. Consequently, residual confounding by indication for use is reduced. In light of this, our results provide some evidence that there is most likely no causal link between the timing of prenatal analgesic opioid exposure and ADHD, both as symptoms and diagnosis.

The most reported substance among opioid exposed women was the combined product of codeine and paracetamol, and our results are most representative for this substance. Therefore, disentangling the sole association of opioids may be challenging. We tried to address this by excluding women using the combined product in a sensitivity analysis, and we found no associations with ADHD or symptoms among children with vs without exposure. Prior studies^43,44^ have reported a positive association between paracetamol use during pregnancy and ADHD in children, showing HRs of a magnitude of 1.37 (95% CI, 1.19-1.59) for receiving a diagnosis of hyperkinetic disorder following ever exposure in pregnancy and an HR of 2.20 (95% CI, 1.50-3.24) for ADHD diagnosis following exposure to paracetamol for more than 29 days during pregnancy. Whether this association is causal or due to bias is a debated topic.^45,46^ The combined product of paracetamol and codeine may be used under circumstances that are more heterogeneous than stronger opioids, and our latter finding may indicate that confounding might play a larger role on our findings.

Longer duration of use may be indicative of a more severe pain condition. We tried to address this by including number of reported pain episodes in our models as a proxy of pain severity. We acknowledge the lack of data of how severe this pain was, and we cannot rule out unmeasured confounding by pain severity.

ADHD and its symptoms are highly heritable,^18,47^ and we tried to address this by including information about proxies of parental ADHD (maternal and paternal filled prescriptions for ADHD medications) in all models and maternal ADHD traits in a subsample. This did not substantially change our main estimates; however, we cannot exclude the role of unmeasured genetic factors in this study.^48^

To evaluate unmeasured confounding, we found that an E value of 2.58 was required to explain our association in the duration analysis. Leppert et al^48^ discuss several early-life exposures (eg, smoking, nutritional supplements, stressful life events, toxin exposure, infections, and more) associated with neurodevelopmental risk alleles. None of these factors was of an order of magnitude that could account for our observed association. However, we cannot exclude a possible role of residual or unmeasured confounding on our results.

If the association between prenatal analgesic opioid exposure and ADHD were causal, we would have expected a higher proportion of children displaying ADHD symptoms at age 5 years and a positive association in our positive control analysis of opioid-containing cough medications. However, we cannot rule out that loss of follow-up in the MoBa study have affected our findings on ADHD symptoms. Future studies on the long-term safety of analgesic opioids should include measures of dose and pain severity and include more domains of neurodevelopment, including cognition.

### Limitations

This study has limitations. The MoBa study has a moderate participation rate (41%), with a possibility of self-selection of the healthiest women into the cohort.^23^ Although association measures have been shown to be valid in MoBa in relation to immediate birth outcomes,^49,50^ we cannot rule the impact of selection bias on our results regarding ADHD symptoms when children were aged 5 years. Furthermore, the ADHD symptoms were parent reported,^51^ and although outcome misclassification cannot be ruled out, this was probably nondifferential. Also, the internal consistency of the CPRS-R is high (Cronbach α = 0.9). Due to low sample size, it was not possible to study the associations of individual opioids. We were not able to identify whether a clear duration association was in place due to low power, which prevented us from looking at more granular duration groups. Another limitation is that we did not have information regarding dosage or duration of use of opioids in MoBa. A mother who had reported use of opioids during one 4-week interval may have used the drug only once or possibly every day. However, mothers who reported use during 2 or more 4-week periods (ie, ≥5 weeks) are more likely to have consumed a higher total dose. This assumption is supported by results from the DDD analysis that showed that mothers who reported opioids in 4 or fewer weeks were dispensed an average of 8.6 DDD, and mothers who reported use in 5 or more weeks were dispensed on average 37.2 DDD. However, any conclusions with regard to duration should be interpreted with caution, as this is representative for only a subsample and not all prescribed medications are actually taken.^52^

## Conclusions

In this cohort study, we found no associations between the timing of analgesic opioid exposure during pregnancy and ADHD, both as diagnosis and symptoms. The risk of ADHD was slightly increased after exposure for 5 or more weeks compared with exposure for 4 or fewer weeks. This result may be associated with longer duration of use, but we cannot exclude the potential role of residual or unmeasured confounding. This finding needs to be replicated in other studies. Adequate pain management in pregnancy should be discussed on an individual patient level, bearing in mind the benefits and risks of different analgesic therapies.

## Notes

Supplement. **eMethods.** Supplemental Methods **eTable 1.** Included Pain Conditions Relevant for Opioid Use in MoBa **eTable 2.** Use of Specific Opioids During Pregnancy **eTable 3.** Specification of Various Treatment Models **eTable 4.** Characteristics of Generated Weights By Exposure in Main Analysis **eTable 5.** Characteristics of Pregnancies in the ADHD Symptoms Sample According to Exposure Status **eTable 6.** Incidence Rate of ADHD by Child Age Bands and Exposure Group **eTable 7.** Association Between Timing of Prenatal Analgesic Opioid Exposure and ADHD Diagnosis in a Subsample With Data From 2004 to 2008 **eTable 8.** Association Between Duration of Prenatal Analgesic Opioid Exposure and ADHD Diagnosis in a Subsample With Data From 2004 to 2008 **eTable 9.** Crude and Weighted Hazard Rate of ADHD According to the Various Exposure Definitions and by Child Age Band **eFigure 1.** Timeline Showing Coverage of the Different Data Sources **eFigure 2.** Simplified Directed Acyclic Graph Showing Assumed Covariate Structure **eFigure 3.** Associations of Timing of Analgesic Opioid Exposure in Pregnancy With ADHD Compared With Unexposed in Time Window and With Prepregnancy Use Only Under Main and Alternative Model Specifications **eFigure 4.** Associations of Timing of Analgesic Opioid Exposure in Pregnancy With ADHD Symptoms Among Children Aged 5 Years Compared With Unexposed in Time Window and With Prepregnancy Use Only Under Main and Alternative Model Specifications **eFigure 5.** Associations of Length of Analgesic Opioid Exposure in Pregnancy With ADHD and ADHD Symptoms in Children Aged 5 Years Under the Main and Alternative Model Specifications **eFigure 6.** Crude and Weighted Nelson-Aalen Cumulative Hazard Estimate Curves Showing the Estimated Proportion of Children Receiving ADHD Diagnoses by Timing of Exposure to Analgesic Opioids in Pregnancy Compared With Unexposed in Time Window and Prepregnancy Use Only **eFigure 7.** Crude and Weighted Nelson-Aalen Cumulative Hazard Estimate Curves Showing the Estimated Proportion of Children Receiving ADHD Diagnoses by Length of Exposure to Analgesic Opioids in Pregnancy
eReferences.
