# Supplementary material for: Association of Timing and Duration of Prenatal Analgesic Opioid Exposure With Attention-Deficit/Hyperactivity Disorder in Children
Source: JAMA Netw Open. 2021 Sep 15;4(9):e2124324. doi: 10.1001/jamanetworkopen.2021.24324 (PMC8444023; doi:10.1001/jamanetworkopen.2021.24324)
Supplement: Supplement. — eMethods. Supplemental Methods eTable 1. Included Pain Conditions Relevant for Opioid Use in MoBa eTable 2. Use of Specific Opioids During Pregnancy eTable 3. Specification of Various Treatment Models eTable 4. Characteristics of Generated Weights By Exposure in Main Analysis eTable 5. Characteristics of Pregnancies in the ADHD Symptoms Sample According to Exposure Status eTable 6. Incidence Rate of ADHD by Child Age Bands and Exposure Group eTable 7. Association Between Timing of Prenatal Analgesic Opioid Exposure and ADHD Diagnosis in a Subsample With Data From 2004 to 2008 eTable 8. Association Between Duration of Prenatal Analgesic Opioid Exposure and ADHD Diagnosis in a Subsample With Data From 2004 to 2008 eTable 9. Crude and Weighted Hazard Rate of ADHD According to the Various Exposure Definitions and by Child Age Band eFigure 1. Timeline Showing Coverage of the Different Data Sources eFigure 2. Simplified Directed Acyclic Graph Showing Assumed Covariate Structure eFigure 3. Associations of Timing of Analgesic Opioid Exposure in Pregnancy With ADHD Compared With Unexposed in Time Window and With Prepregnancy Use Only Under Main and Alternative Model Specifications eFigure 4. Associations of Timing of Analgesic Opioid Exposure in Pregnancy With ADHD Symptoms Among Children Aged 5 Years Compared With Unexposed in Time Window and With Prepregnancy Use Only Under Main and Alternative Model Specifications eFigure 5. Associations of Length of Analgesic Opioid Exposure in Pregnancy With ADHD and ADHD Symptoms in Children Aged 5 Years Under the Main and Alternative Model Specifications eFigure 6. Crude and Weighted Nelson-Aalen Cumulative Hazard Estimate Curves Showing the Estimated Proportion of Children Receiving ADHD Diagnoses by Timing of Exposure to Analgesic Opioids in Pregnancy Compared With Unexposed in Time Window and Prepregnancy Use Only eFigure 7. Crude and Weighted Nelson-Aalen Cumulative Hazard Estimate Curves Showing the Estimated Proportion of Childr [file jamanetwopen-e2124324-s001.pdf]

Supplemental Online Content

Trønnes JN, Lupattelli A, Handal M, Skurtveit S, Ystrom E, Nordeng H. Association of timing and duration of prenatal analgesic opioid exposure with attention-deficit/hyperactivity disorder in children. *JAMA Netw Open.* 2021;4(9):e2124324. doi:10.1001/jamanetworkopen.2021.24324

eMethods. Supplemental Methods

eTable 1. Included Pain Conditions Relevant for Opioid Use in MoBa

eTable 2. Use of Specific Opioids During Pregnancy

eTable 3. Specification of Various Treatment Models

eTable 4. Characteristics of Generated Weights By Exposure in Main Analysis

eTable 5. Characteristics of Pregnancies in the ADHD Symptoms Sample According to Exposure Status

eTable 6. Incidence Rate of ADHD by Child Age Bands and Exposure Group

eTable 7. Association Between Timing of Prenatal Analgesic Opioid Exposure and ADHD Diagnosis in a Subsample With Data From 2004 to 2008

eTable 8. Association Between Duration of Prenatal Analgesic Opioid Exposure and ADHD Diagnosis in a Subsample With Data From 2004 to 2008

eTable 9. Crude and Weighted Hazard Rate of ADHD According to the Various Exposure Definitions and by Child Age Band

eFigure 1. Timeline Showing Coverage of the Different Data Sources

eFigure 2. Simplified Directed Acyclic Graph Showing Assumed Covariate Structure

eFigure 3. Associations of Timing of Analgesic Opioid Exposure in Pregnancy With ADHD Compared With Unexposed in Time Window and With Prepregnancy Use Only Under Main and Alternative Model Specifications

eFigure 4. Associations of Timing of Analgesic Opioid Exposure in Pregnancy With ADHD Symptoms Among Children Aged 5 Years Compared With Unexposed in Time Window and With Prepregnancy Use Only Under Main and Alternative Model Specifications

eFigure 5. Associations of Length of Analgesic Opioid Exposure in Pregnancy With ADHD and ADHD Symptoms in Children Aged 5 Years Under the Main and Alternative Model Specifications

eFigure 6. Crude and Weighted Nelson-Aalen Cumulative Hazard Estimate Curves Showing the Estimated Proportion of Children Receiving ADHD Diagnoses by Timing of Exposure to Analgesic Opioids in Pregnancy Compared With Unexposed in Time Window and Prepregnancy Use Only

eFigure 7. Crude and Weighted Nelson-Aalen Cumulative Hazard Estimate Curves Showing the Estimated Proportion of Children Receiving ADHD Diagnoses by Length of Exposure to Analgesic Opioids in Pregnancy

eReferences.

This supplemental material has been provided by the authors to give readers additional information about their work.

© 2021 Trønnes JN et al. *JAMA Network Open.*

eMethods. Supplemental Methods

Complete MoBa questionnaires are available at: www.fhi.no/en/studies/moba/for-forskere-artikler/questionnaires-from-moba

Additional details on «Methods»

Exposure

The table below shows patterns of exposure to analgesic opioids during pregnancy.

Exposure pattern	ADHD diagnosis sample<br>N total =73 480<br>n (%)	ADHD symptoms sample<br>N total=31 270<br>n (%)
Exposed pre-pregnancy only	838 (1.2)	334 (1.1)
Exposed during pregnancy	1726 (2.3)	667 (2.1)
“Continuers”	402 (0.5)	149 (0.5)
“Initiators”	1324 (1.8)	518 (1.7)
Exposed in early pregnancy only[a]	341 (0.5)	124 (0.4)
Exposed in mid/late pregnancy only[b]	837 (1.1)	337 (1.1)

a) Unexposed pre-pregnancy and in 2nd and 3rd trimester
b) Unexposed pre-pregnancy and in 1st trimester

Outcome

We examined childhood ADHD using both a parent-reported ADHD symptoms scale and ADHD diagnosis and/or filled prescriptions for ADHD medications. The first may capture more subtle daily problems noticed by parents not reaching the threshold of a clinical diagnosis, whereas ADHD diagnosis is indicative of a certain level of severity.[1]

ADHD symptoms: Parent-reported symptoms of ADHD in children at five years of age were measured by 12 items from the Conners Parent Rating Scale-Revised Short Form (CPRS-R(S)) included in the MoBa questionnaire at five years.[2,3] CPRS is a tool for obtaining parental reports of childhood behavior problems, and the 12 selected items are from the areas of inattention and hyperactivity/impulsivity. Parents reported how much each item has been a problem for the child during the past month. The items are scored on a four point Likert scale (1-4) ranging from “not true/seldom” to “very often”. Mean scores were calculated and standardized. Higher z-scores indicated more symptoms of ADHD. The Cronbach’s α for the CPRS-R was 0.9, showing good internal consistency between items of the scale. For this analysis, the ADHD diagnosis sample was further restricted to those with available outcome data in MoBa Q-5years.

© 2021 Trønnes JN et al. *JAMA Network Open.*

Potential confounding factors

The table below shows the source of the variables in the analyses and how they were handled.

Variables	Source	Comments
SES and lifestyle factors		
Maternal age at delivery	MBRN	Continuous
Paternal age	MoBa Q-father	Categorical, 4 categories
Marital status	MBRN	Categorical, 2 categories
Parity	MBRN	Categorical, 2 categories
Education level	MoBa Q1 / Q-father	Categorical, 2 categories<br><br>Education level was coded as the highest level of completed or ongoing education and classified as either:<br>i) high school or lower; or<br>ii) university/college.<br>Coded separately for mothers and fathers.
Maternal income	MoBa Q1	Categorical, 3 categories
Pre-pregnancy BMI	MoBa Q1	Continuous
Folate intake	MoBa Q1	Categorical, 2 categories<br><br>Folic acid supplement was coded as folic acid intake before and during pregnancy (4 weeks prior to pregnancy and/or until week 12 of pregnancy).
Smoking habits	MoBa Q1	Categorical, 3 categories
Alcohol use	MoBa Q1	Categorical, 3 categories
Symptoms of anxiety and depression	MoBa Q1	Continuous (z-score)<br><br>Symptoms of anxiety/depression were measured by a short version of the Hopkins Symptoms Checklist (SCL) at gestational week 17[4] and includes five questions. The SCL is a psychometric scale designed to screen for symptoms of depression in population surveys.[5] Mean summary scores were calculated and standardized to have a mean of zero and a standard deviation of one.
Co-medications	MoBa Q1, Q3, and Q4	Categorical.<br>Separate categories for paracetamol, triptans, antiepileptics, anti-psychotics, anti-depressants, NSAIDs, and benzodiazepines and benzodiazepine-like drugs.
Illicit drug use	MoBa Q1	Categorical, 2 categories
Episodes of pain	MoBa Q1, Q3	Categorical, 1-13<br>In order to account for pain severity, we counted the number of different pain conditions a woman reported during pregnancy in the questionnaires, ranging from 1-13 (see eTable 1).
Maternal chronic conditions	MoBa Q1	Categorical, 2 categories<br>Chronic conditions at baseline were defined if a pregnant woman reported asthma, diabetes, hypertension, arthritis, lupus, Crohn's disease, epilepsy, multiple sclerosis or cancer in the first MoBa questionnaire.
Parental ADHD medication	NorPD	Categorical, 2 categories<br>Separate for mothers and fathers.
ASRS	MoBa Q-3years and Q-father	Categorical, 3 categories<br><br>Mothers were asked to complete the short form of the "Adult ADHD Self-Report Scale (ASRS) in Q6 (child

© 2021 Trønnes JN et al. *JAMA Network Open.*

		age 3 years). ASRS is a self-report screening scale of adult ADHD and includes six questions. Four questions capture symptoms of inattention and two questions entail symptoms of hyperactivity - impulsivity. The five response options range from “never=1” to “very often=5”. We dichotomized the response options (≤ 3=0, ≥ 4=1) and summed them across the six questions, as indicated in Kessler et al.[6] We then categorized the final ASRS score into three clinical categories as recommended (no ADHD symptoms=score 0-1, mild symptoms=score 2-3, moderate to severe symptoms=score 4-6). Due to a large amount of missing in the maternal ASRS (41.6% in the ADHD diagnosis sample, and 18.4% in the ADHD symptoms sample), we used this variable only in a sub-analysis.
Child characteristics		
Child gender	MBRN	Categorical, 2 categories
Malformations	MBRN	Categorical, 2 categories
Prematurity	MBRN	Categorical, 2 categories

SES, Socioeconomic status; MBRN, The Medical Birth Registry of Norway; MoBa, The Norwegian Mother, Father and Child Cohort Study, NorPD; Norwegian Prescription Database; ASRS, Adult ADHD Self-Report Scale.

Sensitivity analyses

We performed many sub-group and sensitivity analysis to test the robustness of our findings.

We conducted separate models for all exposure definitions that took into account additional paternal and child factors under alternate model specifications (eTable 3).

We descriptively examined the cumulative incidence of ADHD by prenatal analgesic opioid exposure status among boys and girls (ADHD diagnosis sample) and we performed stratified analysis with ever/never exposure to analgesic opioid exposure in pregnancy in order to better understand the role of child sex on ADHD risk.

We performed a positive control analysis with women using opioid containing cough medications during pregnancy (ATC R05D). We used propensity scores (PS) with inverse probability of treatment weights (IPTW) to account for measured confounders. First, we fit a logistic regression model to estimate the probability of exposure to R05D during pregnancy, relative to unexposed to R05D in pregnancy, conditional on confounders in model 1 (cf eTable 3) and opioid use during pregnancy. Then we performed Cox regression analysis to estimate HR and generalized linear models to estimate standardized mean differences in ADHD symptoms, as described earlier. The following substances are marketed in Norway under R05D: ethylmorphine, hydrocodone, codeine, noscapine, and combinations (cough syrup; ethylmorphine and codeine).[7]

We performed an analysis among women using opioids not in combination with paracetamol. Thus we excluded those who had used the drug with ATC-code N02AA59 (paracetamol + codeine) during pregnancy. PS with IPTW was applied as described earlier.

We calculated the E-value in order to determine how strong an unmeasured confounder had to be in order to explain away the observed exposure-outcome association.[8-10]

We replicated the main analysis in a sub-sample of pregnancy-child dyads born in 2004 or later (ADHD diagnosis sample, n=54 740), so that all children had available outcome data since birth.

We conducted a complete case analysis (ADHD diagnosis sample: n=59 222, and ADHD symptoms sample: n=26 329).

We excluded 644 children (0.8% of the ADHD diagnosis sample) who had emigrated, because the study did not have information about dates of potential emigration or death.

© 2021 Trønnes JN et al. *JAMA Network Open.*

In the time-to-event analysis, the proportional hazard (PH) assumption was not met (timing analysis only). We therefore split the follow-up time at child age 7 years, estimating period-specific HRs. The follow-up time was split at this time-point based on the weighted Nelson Aalen curves (see eFigure 6 and eFigure 7).

In our propensity score (PS) models we adjusted for a range of variables, including anxiety/depression measured at gestational week 17, number of pain episodes throughout pregnancy and co-medications used during pregnancy. We performed additional sensitivity analyses were we 1) removed anxiety/depression and number of pain episodes, from the main PS models, and 2) adjusted for use of co-medications at baseline (MoBa Q1) in order to see if some of these variables could potentially be mediators in the exposure-outcome relationship.

Missing data

ADHD diagnosis sample: In this sample, 19.4% of the women had missing in at least one of the important confounders. Specifically, missing data were found in the following covariates; maternal education (0.4%), income (3.3%), BMI (2.5%), smoking (1.2%), alcohol intake (10.9%), and symptoms of depression/anxiety (SCL-5) (3.5%). Missing values were also found in paternal age (0.3%) and paternal education (1.1%).

ADHD symptoms sample: In this sample, 15.8% of the women had missing in at least one of the important confounders. Specifically, missing data were found in the following covariates; maternal education (0.4%), income (2.6%), BMI (1.8%), smoking (0.5%), alcohol intake (8.6%), symptoms of depression/anxiety (SCL-5) (2.8%), and prematurity (0.4%). Missing values were also found in paternal age (0.2%) and paternal education (0.7%).

Under the assumption that data were missing at random, we imputed incomplete data via multiple imputation with chained equation (ten replications).[11] The imputation procedure included exposure and outcome variables, baseline hazard, and auxiliary variables (e.g., maternal age and illnesses, parity, co-medication, risk factors for the outcomes).[11,12] Imputed data were used in all analyses.

We chose to impute 10 datasets as this number (m=10) was considered to be a good trade-off to address bias, between (a) the computational effort of having a high number of imputations, and (b) the low percentage of incomplete observations in our study (<1 to 11% for individual variables, leading to 19.4% observations having at least one covariate with missing value)

Additional details on «Results»

Sensitivity analyses

The point estimates under alternative model specifications were generally consisted with main findings (eFigure 3-5). We only had data on maternal ADHD traits for a sub-sample of the population. In this sub-sample, the point estimates were generally similar as in the main analysis, except those comparing mid/late exposure to unexposed in the time window and pre-pregnancy users only, respectively.

The cumulative incidence for ADHD was greater among boys than girls in the study (data not shown). In analyses stratified by gender the weighted HR of ADHD was 1.28 (95% CI: 0.93-1.77) among boys and 1.36 (95% CI: 0.74-2.51) among girls. The point estimates were somewhat similar, however, boys seem to be at elevated risk based on the lower bound of the CI. However, girls were likely in a small sample size. Furthermore, we found no association between opioids and ADHD symptoms in children five years of age stratified by gender (Boys: β_w: -0.01, 95% CI -0.13-0.12 and girls: β_w: 0.06, 95% CI: -0.10-0.21).

There were 1153 (1.6%) and 492 (1.6%) women using opioid containing cough medications (R05D) during pregnancy in the ADHD diagnosis sample and the ADHD symptoms sample, respectively. We found no association between use of R05D during pregnancy and ADHD, compared to unexposed in pregnancy (Crude HR: 0.84, 95% CI: 0.59-1.21, $_w$HR: 0.70, 95% CI: 0.47-1.05). It is not entirely clear why we observed an apparently protective effect in this analysis. It may be random or due to a possible "health seeking behavior bias". We found no association between use of R05D during pregnancy and ADHD symptoms at child age five years, compared to unexposed during pregnancy (Crude β: -0.03, 95% CI: -0.12-0.05, β_w: 0.01, 95% CI: -0.10-0.12).

There were 160 (0.22%) and 60 (0.20%) women who had used opioids not containing paracetamol in the ADHD diagnosis sample and the ADHD symptoms sample, respectively. We found no associations with ADHD (Crude HR: 1.47, 95% CI: 0.70-3.10, $_w$HR: 0.59, 95% CI: 0.23-1.53) or ADHD symptoms (Crude β: 0.28, 95% CI: 0.00-0.56, β_w: 0.12, 95% CI: -0.21-0.45) when compared to unexposed during pregnancy. The weighted models were additionally adjusted for i) alcohol and ii) education, income, folate and alcohol, respectively, due to imbalance of

© 2021 Trønnes JN et al. *JAMA Network Open.*

covariates. When pre-pregnancy users only acted as comparator the crude estimate for ADHD was: HR: 0.97, 95% CI: 0.43-2.18 and for ADHD symptoms the crude estimate was: β: 0.15, 95% CI: -0.15-0.45.

The e-value was calculated to be 2.58, which means that confounding of strength equal to 2.58 (on both sides) could completely explain away an observed HR of 1.60 between use of opioids in ≥5 weeks vs use in ≤4 weeks and ADHD, but a weaker confounder could not.

In a subsample with data available in both MoBa and NorPD (n=50 925), the average DDDs reported among women using opioids in ≤4 weeks and ≥5 weeks was 8.6 DDD (SD 8.5) and 37.2 DDD (SD 79.0), respectively. The median was 5 and 12.5 for the two groups, respectively.

The results of analyses restricted to children born in 2004 or later are presented in eTable 7 and eTable 8. The point estimates deviated, by 20-40%, from those in the main analysis. In weighted analysis, exposure in early pregnancy was associated with a borderline increased risk of ADHD when compared to unexposed women (HR: 1.75, 95% CI: 1.01-3.03). In the analysis of duration, the point estimate was higher and showed an increased risk, however, with a wide CI (use in ≥5 weeks versus ≤4 weeks; HR: 2.45 95% CI: 1.32-4.54). Moreover, the point estimates for exposure in mid/late pregnancy indicate a reduction in risk; however, the CI crosses the null.

The complete case analysis showed similar results as the main analysis (data not shown).

Analysis excluding emigrated children did not materially change the point estimates from the main results (data not shown).

The results of the period-specific HR are presented in eTable 9. When compared to pre-pregnancy users only the results align with the main results. However, when compared to unexposed the point estimates deviates to some extent, meaning that the time point > 7 is driving the association. The PH assumption was met in the duration analysis (≥5 weeks vs ≤4 weeks).

Adjusting for a mediator could potentially bias results towards the null. However, we saw little indication of this in our study after having performed sensitivity analyses to specifically explore this issue. The point estimates changed very little (<10%) and in both directions (data not shown). If a mediator effect were in place, we would have expected these point estimates to be consistently higher than those in the main analyses were. Conclusions remain the same as for the main analyses.

© 2021 Trønnes JN et al. *JAMA Network Open.*

eTable 1. Included Pain Conditions Relevant for Opioid Use in MoBa

**Indications**	**MoBa Q1 – GW 17**	**MoBa Q3 – GW 30**
Pelvic girdle pain	x	x
Abdominal pain	x	
Back pain	x	x
Neck and shoulder	x	
Arthritis	x*	
Sciatica	x*	
Fibromyalgia	x*	
Other pains in muscles/joints		x
Migraine	x*	
Other headache	x*	
Headache / migraine		x

* Indicate longer-term conditions, which could be reported in the period 6 months prior to pregnancy in addition to during pregnancy.
MoBa, The Norwegian Mother, Father and Child Cohort Study; GW, Gestational week.

© 2021 Trønnes JN et al. *JAMA Network Open.*

eTable 2. Use of Specific Opioids During Pregnancy

**Substance (ATC code)**	**ADHD diagnosis sample, $N_{exposed}$=1726 n (%)**	**ADHD symptoms sample, $N_{exposed}$=667 n (%)**
Morphine (N02AA01)	83 (4.8)	33 (4.9)
Oxycodone (N02AA05)	8 (0.5)	6 (0.9)
Codeine, combinations excluding psycholeptics (N02AA59)	1558 (90.2)	604 (90.6)
Ketobemidone (N02AB01)	58 (3.4)	28 (4.1)
Pethidine (N02AB02)	54 (3.1)	23 (3.4)
Buprenorphine (N02AE01)	5 (0.3)	<5
Ketobemidone and antispasmodics (N02AG02)	23 (1.3)	7 (1.0)
Tramadol (N02AX02)	47 (2.7)	11 (1.6)

ADHD, Attention-Deficit/Hyperactivity Disorder; ATC, Anatomical Therapeutic Chemical Classification

© 2021 Trønnes JN et al. *JAMA Network Open.*

eTable 3. Specification of Various Treatment Models

	Model	Description
**Main**	1	**sIPTW:**<br>**Numerator:**<br>Probability of exposure.<br>**Denominator:**<br>Maternal age, marital status, parity, maternal education, income, pre-pregnancy BMI, folic acid supplement, smoking, alcohol, illicit drug use, symptoms of anxiety and depression at week 17 (SCL-5), co-medications any time during pregnancy, chronic health conditions at baseline, episodes of pain in pregnancy, maternal and paternal filled prescriptions for ADHD medication ever in life since 2004.
**Alternative models**	2	**Like in model 1, but adding paternal correlates:**<br>Paternal age, paternal education to the denominator.
	3	**Like in model 1, but adding risk factors for the outcome:**<br>Congenital anomalies, prematurity, and child gender to the denominator.
	4	**Like in model 1, but adding maternal correlates:**<br>Maternal ADHD traits (ASRS) to the denominator.

ADHD, Attention-Deficit/Hyperactivity Disorder; ASRS, Adult ADHD Self-Report Scale; sIPTW: standardized inverse probability of treatment weight; BMI: body mass index (kg/m^2).
Co-medications in pregnancy include: paracetamol, triptans, anti-epileptics, anti-psychotics, anti-depressants, NSAIDs, benzodiazepines, and benzodiazepine-like drugs.

© 2021 Trønnes JN et al. *JAMA Network Open.*

eTable 4. Characteristics of Generated Weights By Exposure in Main Analysis

	ADHD diagnosis sample		ADHD symptoms sample	
	Estimated IPTW		Estimated IPTW	
	Mean (SD)	Min-Max	Mean (SD)	Min-Max
**Timing of exposure**				
Opioid exposed versus unexposed				
Exposed in early pregnancy	1.00 (0.10)	0.02-6.29	1.00 (0.09)	0.03-4.92
Exposed in mid / late pregnancy	1.00 (0.12)	0.02-6.99	1.00 (0.12)	0.02-6.16
Opioid exposed vs pre-pregnancy use only				
Exposed in early pregnancy	1.00 (0.56)	0.49-13.57	1.00 (0.48)	0.47-7.70
Exposed in mid / late pregnancy	1.00 (0.56)	0.49-11.21	0.99 (0.63)	0.44-12.23
**Duration of exposure**				
Exposure ≥ 5 weeks vs ≤ 4 weeks	1.00 (0.39)	0.41-7.47	1.01 (0.52)	0.38-7.32

ADHD, attention Deficit Hyperactivity Disorder; IPTW, inverse probability of treatment weight; SD, standard deviation;

© 2021 Trønnes JN et al. *JAMA Network Open.*

eTable 5. Characteristics of Pregnancies in the ADHD Symptoms Sample (n=31 270) According to Exposure Status

	ADHD symptoms sample		
	Exposure status		
	**Unexposed**<br><br>**n=30 269**	**Exposed**<br><br>**n=667**	**Exposed pre-pregnancy only**<br>**n=334**
**Maternal characteristics**			
Age at time of delivery, mean ± SD	30.3 ± 4.4	30.7 ± 4.5	29.9 ± 4.6
Married/cohabiting, (%)	96.6	96.1	94.3
Primiparous, (%)	47.6	43.5	59.3
University/college education, (%)	74.6	70.3	68.0
Missing, (%)	*0.4*	*0.0*	*0.6*
Gross yearly income,[a] (%)			
Average	70.4	69.6	69.8
Low	13.7	15.1	15.9
High	13.3	11.8	13.2
Missing, (%)	*2.6*	*3.5*	*1.1*
Mean pre-pregnancy BMI (kg/m^2) ± SD	24.0 ± 4.2	24.7 ± 4.5	24.1 ± 4.3
Missing, %	*1.8*	*1.3*	*1.8*
Folic acid supplement, (%)	85.7	85.3	89.8
Smoking,[b] (%)			
No	81.6	74.8	73.7
Yes	5.2	9.0	9.3
Stopped	12.8	15.7	16.5
Missing, (%)	*0.4*	*0.5*	*0.5*
Alcohol intake,[b] (%)			
No or minimal	89.3	87.4	86.2
Low to moderate	2.1	2.3	2.7
Frequent	0.1	0.3	0.0
Missing (%)	*8.5*	*10.0*	*11.1*
Symptoms of anxiety/depression,[c] mean score ± SD	1.2 ± 0.4	1.4 ± 0.5	1.3 ± 0.4
Missing, %	*1.7*	*2.8*	*3.0*
Chronic health conditions,[d] (%)	12.2	20.4	18.9
Co-medications during pregnancy,[e] (%)	54.3	87.3	64.4
Illicit drug use,[b] (%)	0.2	0.8	0.0
ADHD prescriptions,[f] (%)	0.8	1.8	1.5
**Child characteristics**			
Boy, (%)	50.9	49.6	50.6
Preterm (<37 weeks), (%)	4.4	6.0	4.8
Low birthweight (<2500 g), (%)	2.5	3.1	1.2
Malformations, (%)	4.9	5.1	5.7
**Paternal Characteristics**			
Age (years), (%)			
< 25	3.6	2.7	5.4
25-29	22.1	21.7	24.9
30-34	39.5	41.4	35.6
≥ 35	34.6	33.7	34.1
Missing, (%)	*0.2*	*0.5*	*0.0*
University/college education, (%)	56.0	52.3	49.4
Missing, (%)	*0.7*	*0.6*	*1.2*
ADHD prescriptions,[f] (%)	0.7	1.5	1.2

[a)] Average: $17 450-$46 540, low: <$17 450, high: ≥$46 541. [b)] Measured in MoBa Q1. [c)] Measured by a short version of the Hopkins Symptoms Checklist (SCL-5). [d)] Chronic health conditions include: asthma, diabetes, hypertension, Chron's disease, arthritis, lupus, epilepsy, multiple sclerosis or cancer. [e)] Co-medications in pregnancy include: paracetamol, triptans, anti-epileptic drugs, anti-psychotics, antidepressants, NSAIDS and benzodiazepines and benzodiazepine-like drugs. [f)] Indicate filled prescriptions for ADHD medication ever in life since 2004.

© 2021 Trønnes JN et al. *JAMA Network Open.*

eTable 6. Incidence Rate of ADHD by Child Age Bands and Exposure Group

	Unexposed n=70 916		Exposed n=1726		Exposed pre-pregnancy only n=838	
**Child age in years**	**ADHD events n**	**IR per 1000 py (95% CI)**	**ADHD events n**	**IR per 1000 py (95% CI)**	**ADHD events n**	**IR per 1000 py (95% CI)**
0-3	<5	-	<5	-	-	-
3-4	18	0.3 (0.2-0.4)	<5	-	-	-
4-5	47	0.7 (0.5-0.9)	<5	-	<5	-
5-6	151	2.1 (1.8-2.5)	<5	-	<5	-
6-7	228	3.2 (2.8-3.7)	9	5.2 (2.7-10.1)	<5	-
7-8	404	5.9 (5.4-6.5)	20	12.1 (7.8-18.7)	10	12.4 (6.6-23.0)
8-9	392	6.7 (6.0-7.4)	15	10.4 (6.3-17.2)	<5	-
9-10	324	6.8 (6.1-7.5)	17	14.0 (8.7-22.4)	8	13.6 (6.8-27.2)
10-11	219	6.0 (5.3-6.9)	12	12.3 (7.0-21.7)	7	15.9 (7.6-33.4)
>11	297	5.2 (4.7-5.9)	11	6.8 (3.8-12.2)	<5	-

ADHD, attention Deficit Hyperactivity Disorder; IR, incidence rate; CI, confidence interval; py, person years.

© 2021 Trønnes JN et al. *JAMA Network Open.*

eTable 7. Association Between Timing of Prenatal Analgesic Opioid Exposure and ADHD Diagnosis in a Subsample With Data From 2004 to 2008

	**ADHD diagnosis sample**				
	Opioid users vs unexposed				
**Exposure window**	**N**	**Events, n**	**IR per 1000 py**	**Crude HR (95% CI)**	**Weighted HR (95% CI)**
No opioids in early pregnancy	54 201	1312	2.5	Reference	Reference
Opioids in early pregnancy	539	26	4.9	1.96 (1.33, 2.89)	1.75 (1.01, 3.03)
No opioids in mid/late pregnancy	53 853	1301	2.5	Reference	Reference
Opioids in mid/late pregnancy	887	37	4.2	1.65 (1.19, 2.29)	0.80 (0.56, 1.14)
	Opioid users vs pre-pregnancy use only				
**Exposure window**	**N**	**Events, n**	**IR per 1000 py**	**Crude HR (95% CI)**	**Weighted HR (95% CI)**
Opioids pre-pregnancy only	580	20	3.5	Reference	Reference
Opioids in early pregnancy	539	26	4.9	1.44 (0.80, 2.60)	1.38 (0.73, 2.61)
Opioids in mid/late pregnancy	887	37	4.2	1.17 (0.68, 2.01)	0.94 (0.51, 1.71)

ADHD, attention-deficit/hyperactivity disorder; IR, incidence rate; HR, hazard ratio; CI, confidence interval; SD, standard deviation; py, person years.

© 2021 Trønnes JN et al. *JAMA Network Open.*

eTable 8. Association Between Duration of Prenatal Analgesic Opioid Exposure and ADHD Diagnosis in a Subsample With Data From 2004 to 2008

	ADHD diagnosis sample				
**Length of exposure**	**N**	**Events, n**	**IR per 1000 py**	**Crude HR (95% CI)**	**Weighted HR (95% CI)**
Exposed in ≤4 weeks	743	21	2.8	Reference	Reference
Exposed ≥5 weeks	461	30	6.7	2.57 (1.46, 4.53)	2.45 (1.32, 4.54)

ADHD, attention-deficit/hyperactivity disorder; IR, incidence rate; HR, hazard ratio; CI, confidence interval; SD, standard deviation; py, person years.

© 2021 Trønnes JN et al. *JAMA Network Open.*

eTable 9. Crude and Weighted Hazard Rate of ADHD According to the Various Exposure Definitions and by Child Age Band

	**ADHD diagnosis sample**	
	**Opioid users vs unexposed**	
**Exposure window**	**Crude HR (95% CI)**	**Weighted HR (95%CI)**
No opioids in early pregnancy	Reference	Reference
Opioids in early pregnancy		
Child age band <7 years	1.53 (0.76, 3.07)	1.04 (0.45, 2.38)
Child age band ≥ 7 years	1.81 (1.31, 2.52)	1.42 (0.90, 2.24)
No opioids in mid/late pregnancy	Reference	Reference
Opioids in mid/late pregnancy		
Child age band <7 years	1.51 (0.86, 2.69)	0.68 (0.36, 1.30)
Child age band ≥ 7 years	1.82 (1.39, 2.39)	1.49 (1.00, 2.20)
	**Opioid users vs pre-pregnancy users only**	
**Exposure window**	**Crude HR (95% CI)**	**Weighted HR (95%CI)**
Opioids pre-pregnancy only	Reference	Reference
Opioids in early pregnancy		
Child age band <7 years	1.16 (0.42, 3.19)	1.28 (0.43, 3.75)
Child age band ≥ 7 years	1.17 (0.72, 1.89)	1.10 (0.65, 1.83)
Opioids in mid/late pregnancy		
Child age band <7 years	1.17 (0.46, 2.98)	0.99 (0.37, 2.64)
Child age band ≥ 7 years	1.15 (0.74, 1.78)	1.10 (0.67, 1.80)

ADHD, attention-deficit/hyperactivity disorder; HR, hazard ratio; CI, confidence interval.

© 2021 Trønnes JN et al. *JAMA Network Open.*

eFigure 1. Timeline Showing Coverage of the Different Data Sources

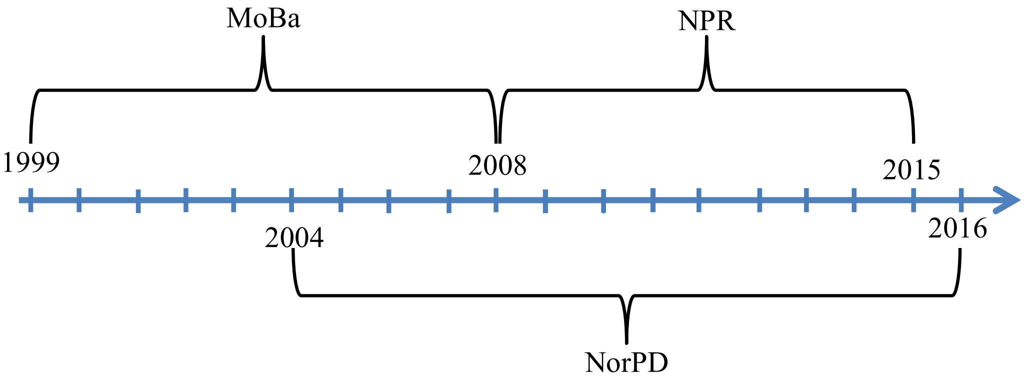

MoBa, The Norwegian Mother, Father and Child Cohort Study; NPR, The Norwegian Patient Registry; NorPD, The Norwegian Prescription Database.

© 2021 Trønnes JN et al. *JAMA Network Open.*

eFigure 2. Simplified Directed Acyclic Graph Showing Assumed Covariate Structure

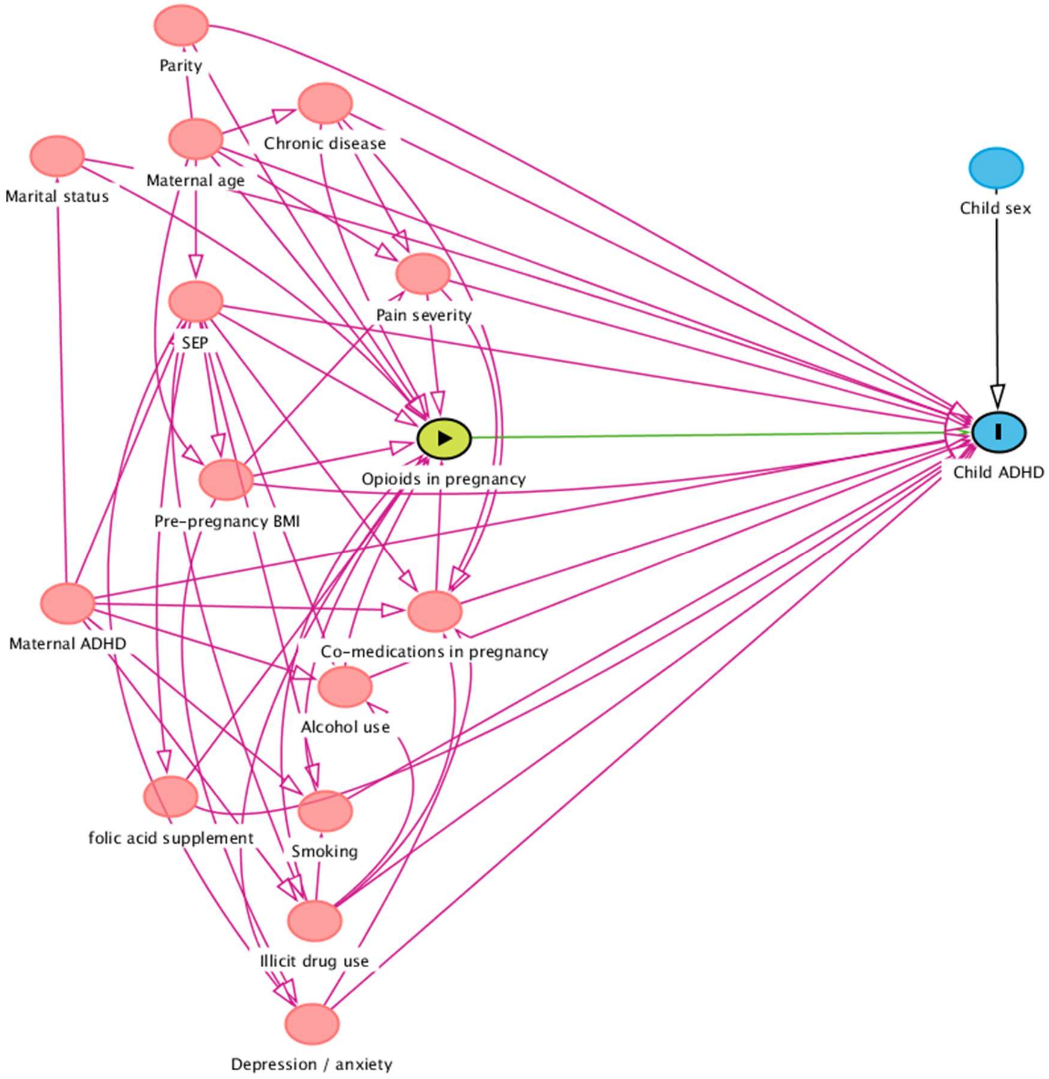

SES, socioeconomic position including maternal income and parental education; ADHD, attention-deficit/hyperactivity disorder.

© 2021 Trønnes JN et al. *JAMA Network Open.*

eFigure 3. **Associations of Timing of Analgesic Opioid Exposure in Pregnancy With ADHD Compared With Unexposed in Time Window (A) and With Prepregnancy Use Only (B) Under Main and Alternative Model Specifications**

a)

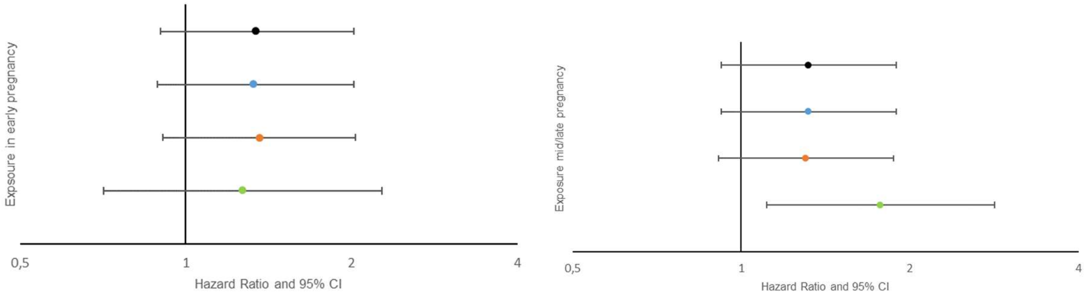

b)

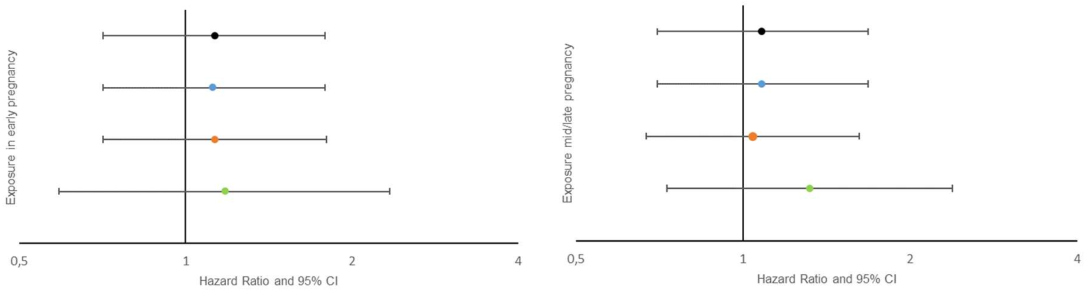

Black = main model

Blue = main model, plus paternal correlates

Orange = main model, plus child characteristics

Green = main model, plus maternal ASRS

© 2021 Trønnes JN et al. *JAMA Network Open.*

eFigure 4. Associations of Timing of Analgesic Opioid Exposure in Pregnancy With ADHD Symptoms Among Children Aged 5 Years Compared With Unexposed in Time Window (A) and With Prepregnancy Use Only (B) Under Main and Alternative Model Specifications

a)

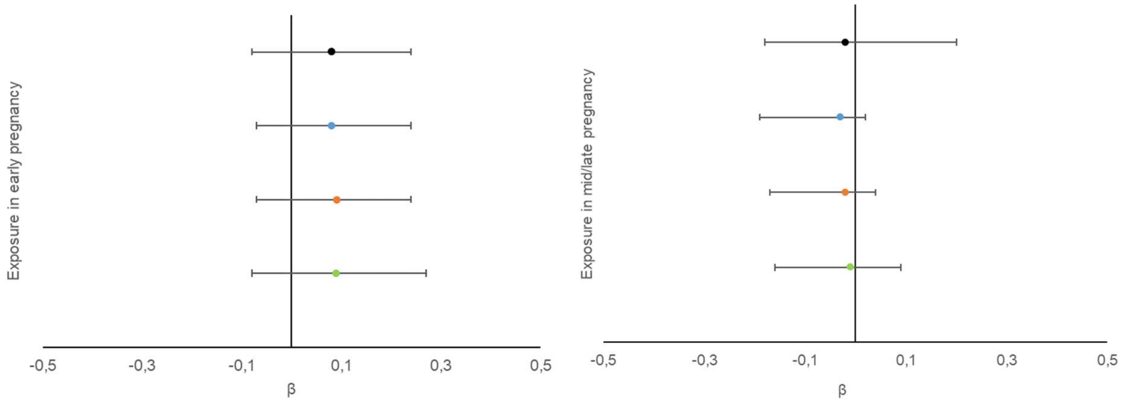

b)

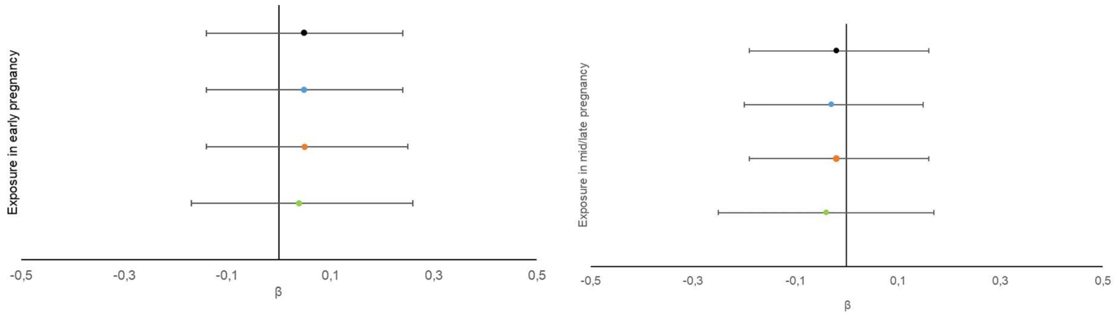

Black = main model

Blue = main model, plus paternal correlates

Orange = main model, plus child characteristics

Green = main model, plus maternal ASRS

© 2021 Trønnes JN et al. *JAMA Network Open.*

eFigure 5. Associations of Length of Analgesic Opioid Exposure in Pregnancy With ADHD (A) and ADHD Symptoms (B) in Children Aged 5 Years Under the Main and Alternative Model Specifications

a)

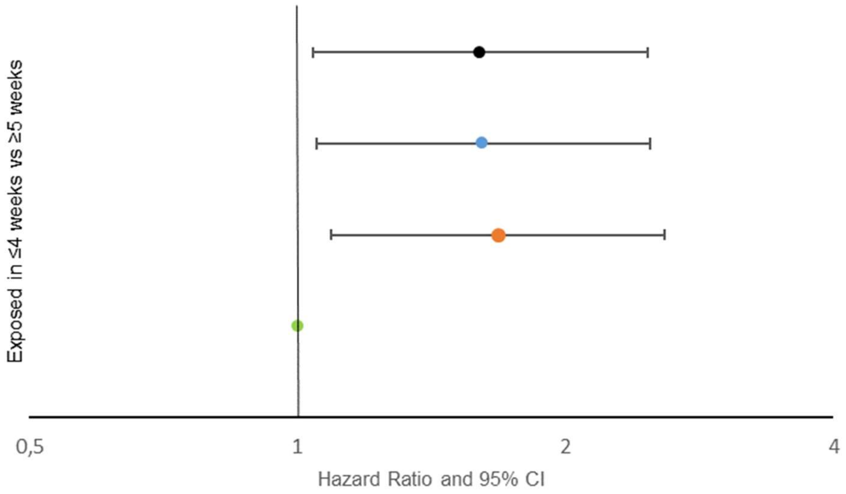

b)

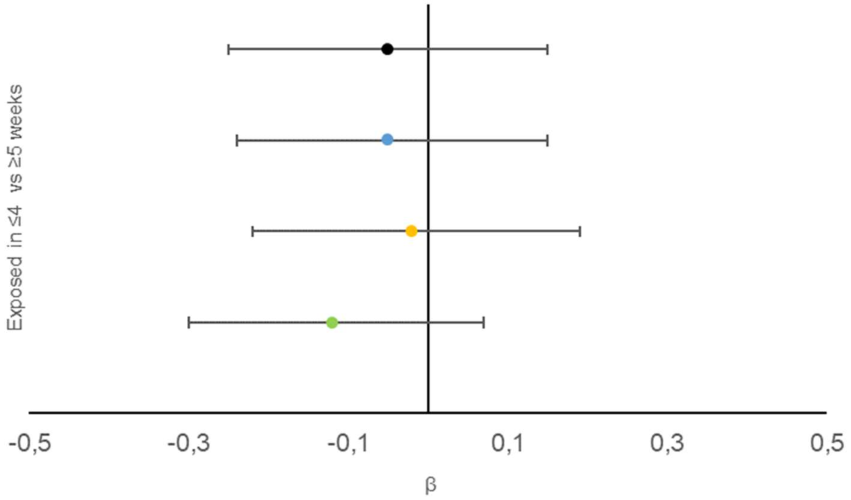

Black = main model

Blue = main model, plus paternal correlates

Orange = main model, plus child characteristics

Green = main model, plus maternal ASRS

© 2021 Trønnes JN et al. *JAMA Network Open.*

eFigure 6. Crude and Weighted Nelson-Aalen Cumulative Hazard Estimate Curves Showing the Estimated Proportion of Children Receiving ADHD Diagnoses by Timing of Exposure to Analgesic Opioids in Pregnancy Compared With Unexposed in Time Window (A) and Prepregnancy Use Only (B)

a)

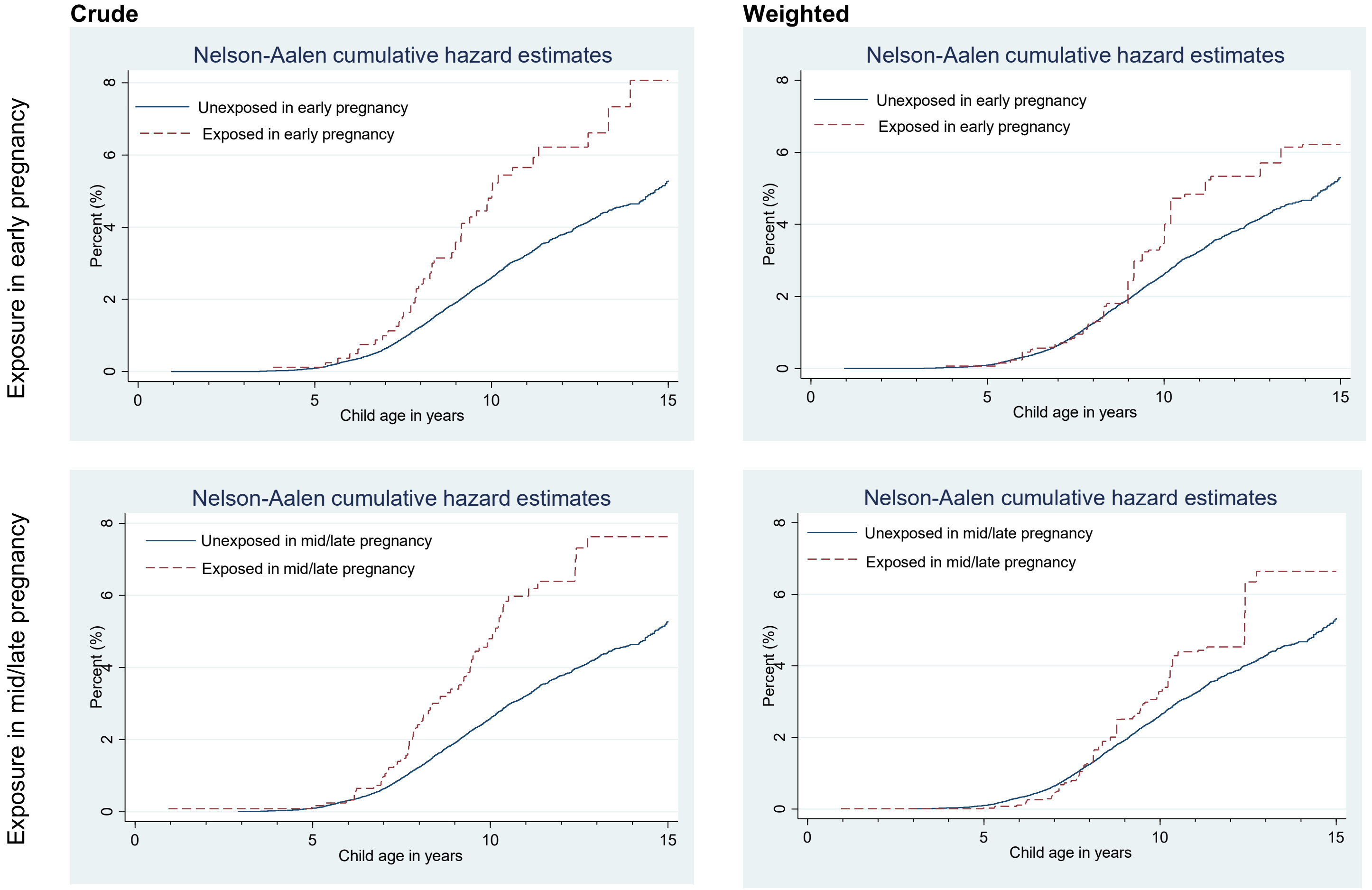

© 2021 Trønnes JN et al. *JAMA Network Open.*

b)

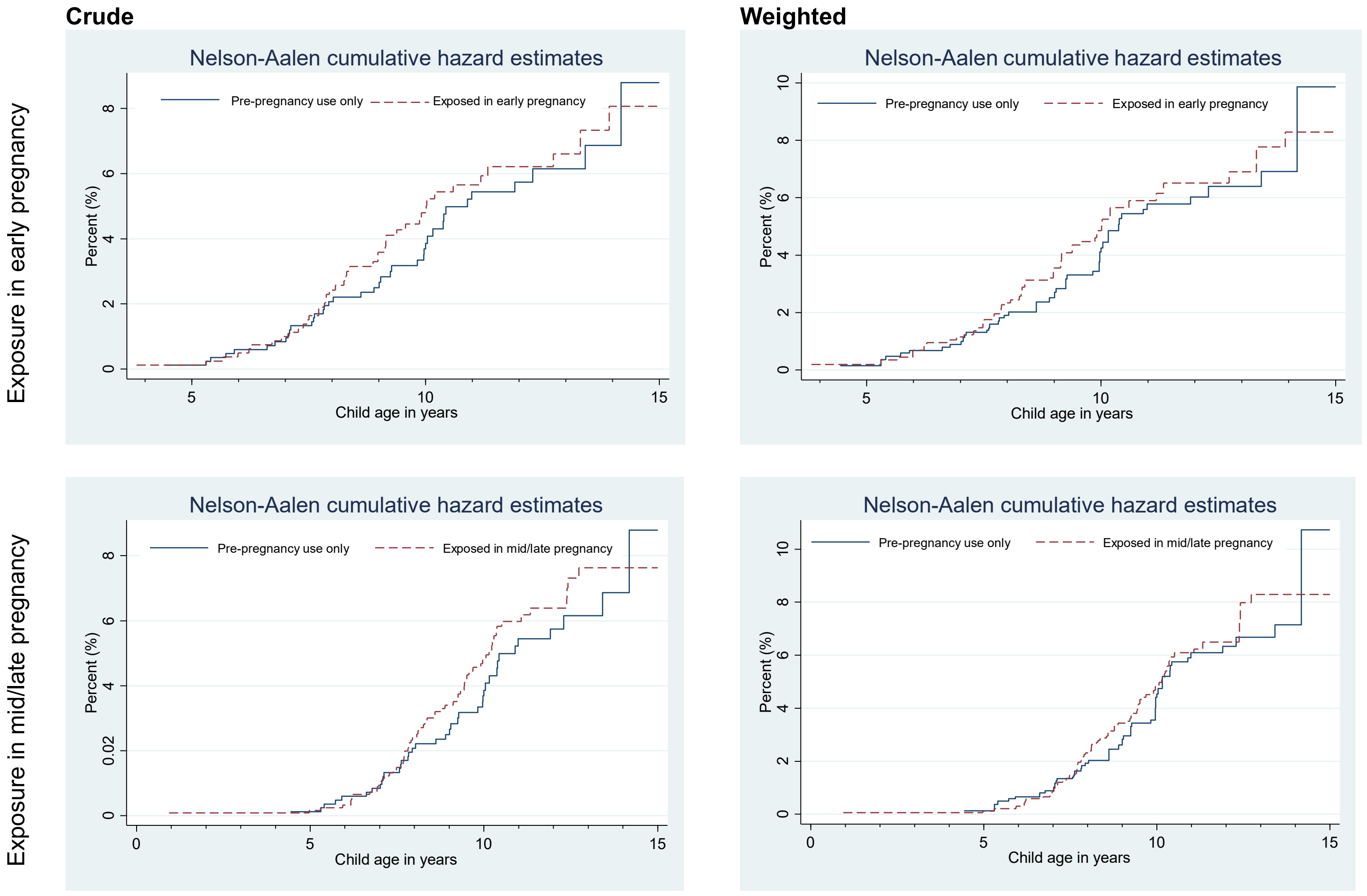

© 2021 Trønnes JN et al. *JAMA Network Open.*

eFigure 7. Crude and Weighted Nelson-Aalen Cumulative Hazard Estimate Curves Showing the Estimated Proportion of Children Receiving ADHD Diagnoses by Length of Exposure to Analgesic Opioids in Pregnancy

a) Crude

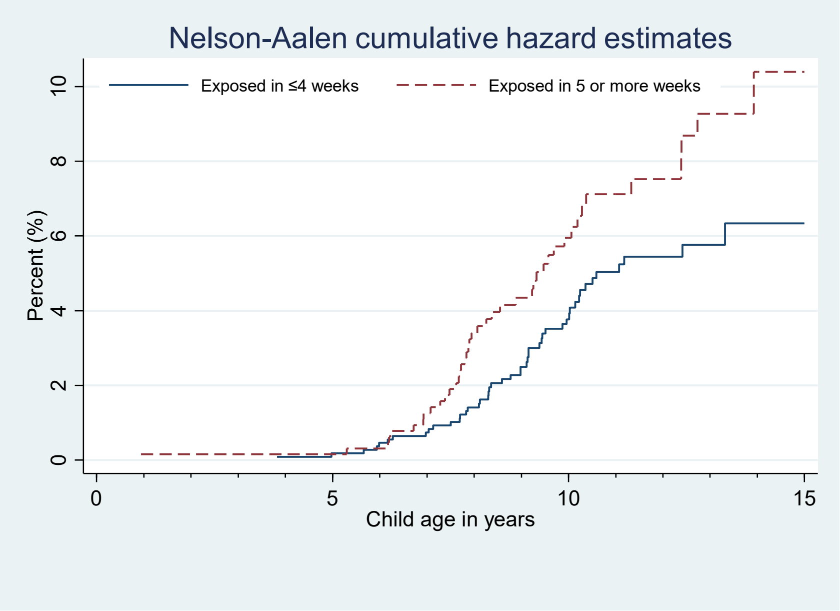

b) Weighted

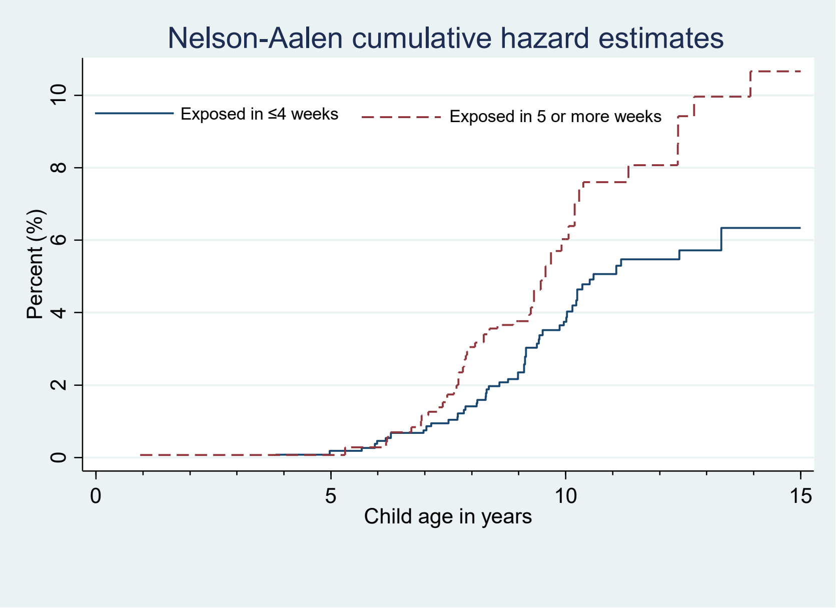

© 2021 Trønnes JN et al. *JAMA Network Open.*

eReferences

1. Hjorth S, Bromley R, Ystrom E, Lupattelli A, Spigset O, Nordeng H. Use and validity of child neurodevelopment outcome measures in studies on prenatal exposure to psychotropic and analgesic medications - A systematic review. *PLoS One.* 2019;14(7):e0219778.

2. Conners CK, Sitarenios G, Parker JD, Epstein JN. The revised Conners' Parent Rating Scale (CPRS-R): factor structure, reliability, and criterion validity. *J Abnorm Child Psychol.* 1998;26(4):257-268.

3. Kumar G, Steer RA. Factorial validity of the Conners' Parent Rating Scale-revised: short form with psychiatric outpatients. *J Pers Assess.* 2003;80(3):252-259.

4. Tambs K, Røysamb E. Selection of questions to short-form versions of original psychometric instruments in MoBa. *Norsk Epidemiologi.* 2014;24(1-2):195-201.

5. Strand BH, Dalgard OS, Tambs K, Rognerud M. Measuring the mental health status of the Norwegian population: a comparison of the instruments SCL-25, SCL-10, SCL-5 and MHI-5 (SF-36). *Nord J Psychiatry.* 2003;57(2):113-118.

6. Kessler RC, Adler L, Ames M, et al. The World Health Organization Adult ADHD Self-Report Scale (ASRS): a short screening scale for use in the general population. *Psychol Med.* 2005;35(2):245-256.

7. FELLESKATALOGEN. https://www.felleskatalogen.no/, Accessed January 7, 2021.

8. E-value calculator. https://www.evalue-calculator.com/, Accessed January 28, 2021.

9. Mathur MB, Ding P, Riddell CA, VanderWeele TJ. Web Site and R Package for Computing E-values. *Epidemiology.* 2018;29(5):e45-e47.

10. VanderWeele TJ, Ding P. Sensitivity analysis in observational research: introducing the E-value. *Ann Intern Med.* 2017;167(4):268-274.

11. Sterne JA, White IR, Carlin JB, et al. Multiple imputation for missing data in epidemiological and clinical research: potential and pitfalls. *BMJ.* 2009;338:b2393.

12. White IR, Royston P. Imputing missing covariate values for the Cox model. *Stat Med.* 2009;28(15):1982-1998.

© 2021 Trønnes JN et al. *JAMA Network Open.*
